# Biosensors for the Determination of Protein Biomarkers

**DOI:** 10.3390/bios13010112

**Published:** 2023-01-09

**Authors:** Zenon Lukaszewski, Ewa Gorodkiewicz

**Affiliations:** 1 Faculty of Chemical Technology, Poznan University of Technology, pl. Sklodowskiej-Curie 5, 60-965 Poznan, Poland; 2 Bioanalysis Laboratory, Faculty of Chemistry, University of Bialystok, Ciolkowskiego 1K, 15-245 Bialystok, Poland

Circulating body fluids such as blood, urea, saliva, cerebrospinal fluid, etc., are potential sources of valuable diagnostic information. Most of these fluids are easily available during routine medical investigations. To date, protein biomarkers have most frequently provided useful information concerning cancer and cardiovascular diseases, while the determination of cancer cells or exosomes in body fluids remains undeveloped [1,2]. Protein biomarkers are very useful diagnostic tools. Many of them, including CA 125, HE4, CEA, and troponins I and T, have established roles in medical diagnostics. Simple blood analyses provide alternatives to biopsy. Therefore, the determination of protein biomarkers in body fluids is called ‘liquid biopsy’. The precise determination of a particular protein biomarker in body fluids makes it possible to determine the stage of a disease and may be helpful in selecting the optimal treatment or therapy. Moreover, post-therapy biomarker determination is used to evaluate the effectiveness of the therapy. The relative ease of protein biomarker determinations in body fluids such as saliva, urea, and blood enables the act of screening a population to discover a disease at an early stage when it is the most easily treatable. The number and effectiveness of currently used protein biomarkers cannot be regarded as satisfactory. Therefore, the discovery of new protein biomarkers is highly desirable.

Biosensors are among the most promising tools used for protein marker determination. An ideal biosensor should be suitable for the determination of a particular protein biomarker in a particular body fluid. It should be highly selective, sufficiently sensitive, precise and accurate, and should not be subject to any hindering effects from the body’s fluids. However, such a perfect biosensor is rarely created in a single stage. Usually, it is developed in several stages, beginning with the conception of the biosensor and followed by analysis of characteristics, such as the range of dynamic responses, precision, accuracy, and potential interferents, and the validation and examples of the biomarker’s determination in real samples. Several stages of readiness or maturity of a biosensor can be distinguished [3]: (i) The biosensor is suitable only for the detection of a protein biomarker; (ii) the biosensor is characterized by basic analytical data under model conditions; (iii) the biosensor is validated and examples of biomarker determination in real samples are given. New applications of existing biosensors are also valuable in terms of the gradual introduction of particular biosensors in analytical practice. In cases with insufficient sensitivity, the analytical signal can be enhanced [4], and the hindering effect of the body’s fluid can be eliminated by the preliminary separation of the determined protein biomarker.
